# A Linkage Disequilibrium–Based Approach to Selecting Disease-Associated Rare Variants

**DOI:** 10.1371/journal.pone.0069226

**Published:** 2013-07-11

**Authors:** Rajesh Talluri, Sanjay Shete

**Affiliations:** 1 Department of Biostatistics, The University of Texas MD Anderson Cancer Center, Houston, Texas, United States of America; 2 Department of Epidemiology, The University of Texas MD Anderson Cancer Center, Houston, Texas, United States of America; University of California, Irvine, United States of America

## Abstract

Rare variants have increasingly been cited as major contributors in the disease etiology of several complex disorders. Recently, several approaches have been proposed for analyzing the association of rare variants with disease. These approaches include collapsing rare variants, summing rare variant test statistics within a particular locus to improve power, and selecting a subset of rare variants for association testing, e.g., the step-up approach. We found that (a) if the variants being pooled are in linkage disequilibrium, the standard step-up method of selecting the best subset of variants results in loss of power compared to a model that pools all rare variants and (b) if the variants are in linkage equilibrium, performing a subset selection using step-based selection methods results in a gain of power of association compared to a model that pools all rare variants. Therefore, we propose an approach to selecting the best subset of variants to include in the model that is based on the linkage disequilibrium pattern among the rare variants. The proposed linkage disequilibrium–based variant selection model is flexible and borrows strength from the model that pools all rare variants when the rare variants are in linkage disequilibrium and from step-based selection methods when the variants are in linkage equilibrium. We performed simulations under three different realistic scenarios based on: (1) the HapMap3 dataset of the DRD2 gene, and CHRNA3/A5/B4 gene cluster (2) the block structure of linkage disequilibrium, and (3) linkage equilibrium. We proposed a permutation-based approach to control the type 1 error rate. The power comparisons after controlling the type 1 error show that the proposed linkage disequilibrium–based subset selection approach is an attractive alternative method for subset selection of rare variants.

## Introduction

Rare variants have increasingly been cited as major contributors to the disease etiology of several complex disorders. A variety of approaches for analyzing rare variants have been proposed [1]–[4]. Morris and Zeggini [4] have shown that tests based on single variants have limited power compared with tests based on summing or collapsing rare variants. Several tests based on collapsing rare variants have been proposed. Originally, Morgenthaler and Thilly [3] proposed the Cohort Allelic Sum Test (CAST) based on the difference in the number of mutant alleles in cases and controls. The Combined Multivariate and Collapsing (CMC) test extends CAST by collapsing all variants below a minor allele frequency threshold but variants above this minor allele frequency threshold are not collapsed and incorporated into multiple regression model [1]. Furthermore, the CMC uses the Hotelling 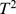 test statistic, which is more robust than the proportion-based test used in the CAST. In both the CMC and CAST, all variants are assumed to have an equal effect on the phenotype. Therefore, Madsen and Browning [2] proposed a Weighted Sum Statistic (WSS), which weighs the variants based on the inverse of the estimated standard deviation of the total number of rare variants in the sample. This strategy assumes that rarer variants have a greater impact on the phenotype. Price et al. [5] proposed a variable allele-frequency threshold method (VT) for selecting rare variants based on the assumption that variants with minor allele frequency below an unknown allele frequency threshold are more likely to be functional. Han and Pan [6] proposed the Sum of Squared Score (SSU) test and its weighted version, which is robust to the direction of the effect of rare variants. Hoffmann et al. [7] used the general regression framework and model the weights as the product of three variables. The multiplicative model allows for weighting, direction of effect, and variant selection to be incorporated into the framework. They also proposed selecting variants based on functional significance and a data-driven method of variable selection called “step-up,” which is based on the standard forward selection algorithm. Recently, Wu et al. [8] proposed a regression approach, sequence kernel association test (SKAT), based on the score-based variance components test.

However, none of these approaches consider information about linkage disequilibrium (LD) among rare variants. In this article, we propose a new approach, based on the LD among rare variants, for selecting a subset of variants to include in the analysis. Using simulations, we compared our subset selection approach to the step-up method of Hoffmann et al. [7], three related variations: the step-down, step-up-down, and step-down-up methods, the VT method, and SKAT. The variants selected using VT approach were analyzed using the logistic regression framework. We considered three different simulation scenarios: (1) a simulation based on the LD structure of the DRD2 gene, and CHRNA3/A5/B4 gene cluster from the HapMap3 dataset [9], (2) a simulation scenario involving LD block simulation, and (3) a scenario which involved simulating the rare variants without any LD structure, that is, all the variants were independent of each other. The power comparisons of the methods in the three scenarios showed that our approach to selecting rare variants based on their LD is an attractive alternative method for selecting rare variants for association studies.

## Methods

We assumed that the data were case-control data from N individuals with a binary phenotype that indicates whether the individuals are affected. Let N_1_ be the number of affected individuals (cases) and N_0_ be the number of unaffected individuals (controls). A locus is a contiguous sequence of common and rare variants in the genome. Our goal was to detect the association of a particular binary phenotype 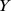 with rare variants at a particular locus *X*.

Let us assume that there are *p* rare variants in the locus of interest. Let 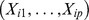 denote the genotype for individual *i*. We choose the collapsing model as detailed in [7]:
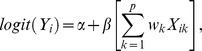
where 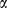 is the intercept, 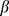 is the regression coefficient of association of the locus with the disease, and 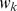 can be modeled as a multiplicative weight, as in



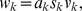
where 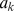 is a continuous weight used to up-weigh the effect of rare alleles based on the minor allele frequency (MAF), 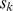 is a binary weight with a value of 1 or −1, depending on the direction of the effect of the variant on the disease (i.e., protective or deleterious), and 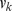 is the binary variable selection weight (0 or 1), which represents whether or not the particular variant *k* is included in the model. Many approaches have been proposed to model 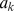 and 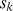 [2], [7]. We concentrated our efforts on selecting the subset of variants that best explain the phenotype based on the available data. Therefore, without loss of generality, we assume 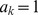 and 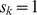, as we are not focusing on optimizing these weights in our model and instead they can be modeled in the most optimal way suggested in the literature [2], [7], [8].

### Step-based Subset Selection Methods

The step-up method [4] is a data-driven method that tries to find the best possible set of rare variants by minimizing the p-value or maximizing a particular test statistic using the standard forward selection algorithm. The forward selection algorithm starts with no variants in the model and, at each iteration, adds variants to the model to maximize the Wald test statistic. The process stops when adding a variant to the model no longer increases the value of the test statistic. Such data-driven greedy algorithms generally do not capture the best model because they optimize the test statistics locally [10]. Typically, the space of all possible models is too large to explore in a reasonable amount of time.

We also tested an alternative version of the step-up method, the backward elimination (step-down) algorithm, which starts with all the variants in the model and removes them until the test statistic is maximized. While the forward selection and backward elimination algorithms are efficient, an even better searching algorithm is step-based selection, which combines both of these steps. Step-based selection alternates between forward selection and backward elimination, allowing the model space to be searched more effectively. Two situations arise in this scenario, one that starts with all the variants in the model and one that starts with no variants in the model (step-down-up and step-up-down, respectively). We explored how these variations fare with the step-up algorithm. The algorithmic details of the implementations for these methods are presented in Text S1.

### Linkage Disequilibrium–based Selection

We performed preliminary simulations to identify rare variants using these step-based approaches and found that (a) when rare variants were in LD, the standard variable selection procedures resulted in a loss of power compared with the model that simply collapses all rare variants (named hereafter the full model) and (b) when rare variants were independent of each other (i.e., no LD among rare variants), step-based variable selection had higher power than the full model. Therefore, we proposed a modification to the variable selection approaches that will account for LD among rare variants. The idea for this approach comes from the fact that including all the rare variants that are in LD with their associated variants enhances the power of the rare variant association.

The proposed algorithm that accounts for LD, called LDSEL, is as follows:

1. Use the step-down-up selection method to select associated variants in the model.
2. For each selected variant in step 1, identify all other variants that are in LD with the selected variants in the cases.
3. The union of variants identified in step 1 and step 2 forms the final selected subset of rare variants.

This model collapses to the regular step-based methods when the rare variants are not in LD. It also collapses to the full model on the other extreme when all the rare variants are in LD with each other. In step 2 of our approach, we used an LD threshold of 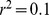 for identifying the variants that are in LD with the associated variants. We performed power comparisons of all the different variable selection approaches referred to above in three simulation scenarios: the HapMap LD scenario, the block LD scenario, and the independent rare variant scenario.

### Simulations

We simulated 1000 replicate samples, each with 1000 cases and 1000 controls, for each of the three scenarios listed below and performed type 1 error and power comparisons based on the simulated data. For the type 1 error comparisons, we generated data from a null model in which none of the variants were causal. The LD structure between the variants was assumed to be from the DRD2 gene of scenario 1. The permutation strategy involved permuting the case-control status and performing the analysis on the permuted data. The permutation-based p value can be estimated by counting the proportion of p-values from the permuted data sets that are less than or equal to the observed p-value for the original dataset. For the type 1 error and power comparisons, 1000 permutations were used for each dataset to obtain the permutation-based p-value.

#### Scenario 1: Simulation from HapMap3 LD structure for the DRD2 gene

In this scenario, we simulated rare variants using the LD structure of the DRD2 gene from the HapMap3 data (HapMap3 Genome Browser, release #2 [Phase 3 - genotypes, frequencies, & LD]). The DRD2 gene spans 112,785,528 bp to 112,851,091 bp on chromosome 11. In total, the HapMap3 database identified 49 variants located in the gene region. However, two of the variants were not polymorphic and so we removed them from further consideration. The pairwise LD pattern (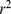) and the MAF information were downloaded from the database. HapSim [11] is a simulation tool for generating haplotype data with pre-specified allele frequencies and LD coefficients. We used HapSim to generate haplotypes for each of the two strands of the DNA based on the LD pattern for the DRD2 gene. The base pairs at each locus are combined without reference to the strand to get the genotype data.

A total of 47 rare variants were simulated. The MAFs of the rare variants were randomly generated from a uniform distribution between 0.25% and 0.5%. Of the 47 variants, 5 variants were randomly designated to be causal variants. A logistic regression model was used to simulate the case-control status.
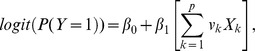
where 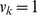, if the variant is causal, and zero otherwise, 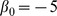 is the log odds of the population disease risk, and 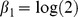 is the log odds ratio for the causal variants.

#### Scenario 1: Simulation from HapMap3 LD structure for the CHRNA3/A5/B4 gene cluster

In order to further evaluate the proposed methodology, we also considered CHRNA3/A5/B4, a gene cluster encompassing multiple genes. The CHRNA3/A5/B4 cluster spans 76,490,686 bp to 76,899,993 bp on chromosome 15. In total, the HapMap3 database identified 186 variants located in the gene region. However, 22 of the variants were not polymorphic and so we removed them from further consideration. The pairwise LD pattern (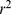) and the MAF information were downloaded from the database. A total of 154 rare variants were simulated. The MAFs of the rare variants were randomly generated from a uniform distribution between 0.25% and 0.5%. Of the 154 variants, 7 variants were randomly designated to be causal variants. A logistic regression model was used to simulate the case-control status as in the previous case.

#### Scenario 2: Simulation from block LD structure

**Figure 1:**
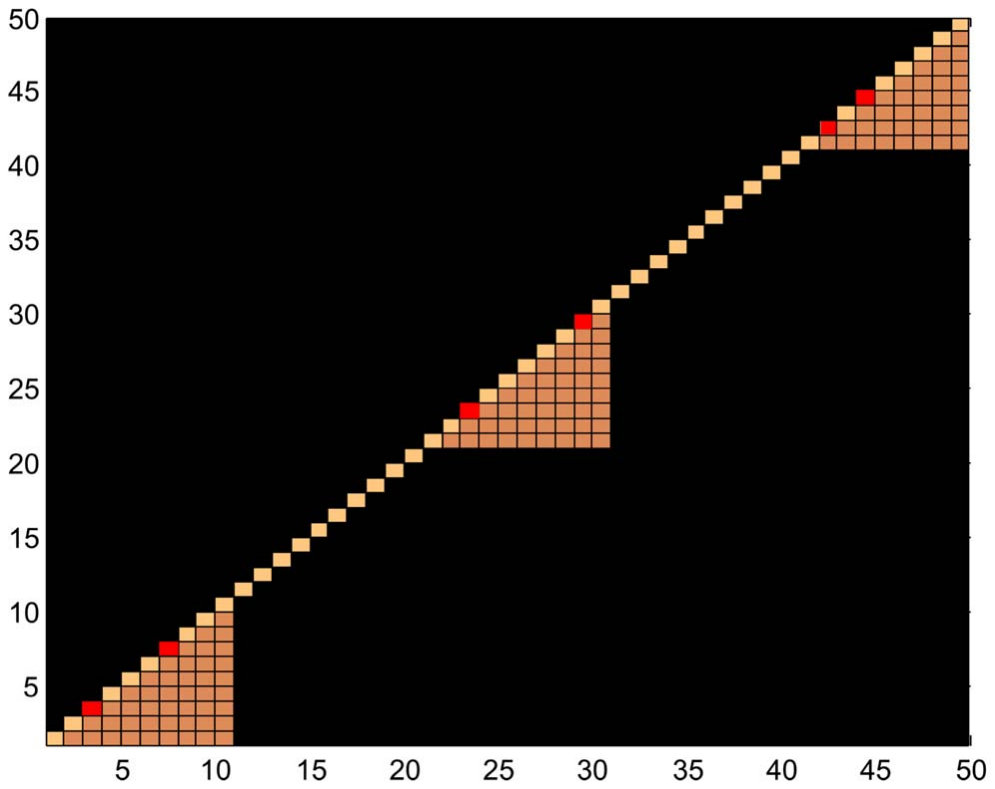
LD structure for block LD simulated data in scenario 2. The orange blocks contain 10 variants each, which are in LD with one another. Two red variants in each block were randomly designated as the causal variants. The rest of the variants are independent of all the other variants.

The simulations for the block LD scenario involved modeling the LD structure of the rare variants as blocks. As in the previous scenario, the MAFs of the rare variants were randomly generated from a uniform distribution between 0.25% and 0.5%. The LD structure for rare variants was simulated from a three-block diagonal structure, as shown in Figure 1. Each LD block had 10 variants that were in LD. Within each block, two variants were randomly designated to be causal. Because of LD, the other 8 variants within each LD block were also associated with the disease. In addition to the 30 associated variants within the three blocks, we simulated 20, 70, or 170 independent variants (i.e., not in LD with any other variants) outside the three LD blocks. These three different numbers (20, 70 and 170) of non-causal and non-associated variants were simulated to assess the performances of the different methods over a range of signal-to-noise ratios. The disease model was the same as in the previous simulation scenario.

#### Scenario 3: Simulation from No LD structure

The no LD scenario involved simulating 50 rare variants that were in linkage equilibrium. Once again, the MAF of each rare variant was randomly generated from a uniform distribution between 0.25% and 0.5%. Of the 50 rare variants, 5 were randomly designated to be causal. The disease model was the same as in the previous simulations.

## Results

We analyzed the 1000 null replicates using the step-up, full model, step-down, step-up-down, step-down-up, LDSEL, SKAT, and VT methods. Table 1 shows the type 1 error values at the 5% and 1% levels of significance for each of these methods. The uncorrected type 1 errors were liberal for all methods except the full model and SKAT. The step-based methods over-fit the data, as they are driven by minimizing the p-value. Therefore, the type 1 error must be controlled using a permutation strategy. As we can see from the second panel of Table 1, the permutation-based corrected type 1 errors were well controlled at the 5% and 1% significance levels for all the methods. For example, the step-up approach had uncorrected type 1 errors of 0.996 and 0.946 at the 5% and 1% levels of significance, respectively, but after permutation-based correction, the type 1 errors were well controlled at 0.052 and 0.009, respectively. The proposed LDSEL method had uncorrected type 1 errors of 0.353 and 0.183 at the 5% and 1% levels of significance, and the corrected type 1 errors for LDSEL were 0.049 and 0.010 for the 5% and 1% levels of significance, which are well controlled. This shows that the permutation-based strategy accurately accounts for over-fitting of the data that results from the subset selection procedure.

**Table 1:** Uncorrected and permutation-based corrected type 1 errors for different methods at the 5% and 1% level of significance.

Method	Type 1 error					
	Uncorrected			Corrected		
	5%		1%	5%		1%
Step-up	0.996	0.946		0.052	0.009	
Full model	0.049	0.012		0.049	0.012	
Step-down	1	0.966		0.051	0.010	
Step-up-down	0.996	0.946		0.052	0.009	
Step-down-up	1	0.966		0.051	0.010	
LDSEL	0.353	0.183		0.049	0.010	
SKAT	0.045	0.011		0.048	0.009	
VT	0.071	0.016		0.053	0.012	

Power comparison for different approaches at the 5% and 1% levels of significance for scenario 1 are presented in Table 2. The first panel corresponds to simulation scenario 1 using the LD structure of the DRD2 gene and the second panel corresponds to simulation scenario 1 using the LD structure of the CHRNA3/A5/B4 gene cluster. All the step-based methods had very similar power. In the first panel, the full model had a power of 0.538 at the 5% level of significance, which was slightly higher than the power for the step-based methods. This is due to the inability of the step-based approaches to select variants in LD with the associated variants which is also reflected in the second panel with a larger cluster of genes. In the first panel, the LDSEL approach had a power of 0.552 at the 5% level of significance, which was higher than SKAT, VT, and the step-based methods. In the second panel, which corresponds to a larger gene cluster, the LDSEL approach had higher power than the step-based methods and VT, but had similar power as the full model and SKAT. The VT method had slightly lower power than the step-based methods in the first panel whereas in the second panel it had higher power than the step-based methods.

**Table 2:** Power comparison for different approaches at the 5% and 1% levels of significance.

Method	Power			
	DRD2		CHRNA3/A5/B4	
	5%	1%	5%	1%
Step-up	0.526	0.297	0.352	0.148
Full model	0.538	0.302	0.565	0.32
Step-down	0.528	0.298	0.359	0.152
Step-up-down	0.526	0.297	0.352	0.148
Step-down-up	0.528	0.298	0.359	0.152
LDSEL	0.552	0.308	0.569	0.329
SKAT	0.468	0.228	0.579	0.323
VT	0.512	0.239	0.486	0.253

The first panel corresponds to simulation scenario 1 using the LD structure of the DRD2 gene and the second panel corresponds to simulation scenario 1 using the LD structure of the CHRNA3/A5/B4 gene cluster.

Table 3 reports the power comparisons for 3 types of datasets simulated using the block LD structure as presented in scenario 2; the three panels in Table 3 correspond to three datasets simulated using 20 non-associated variants, 70 non-associated variants, and 170 non-associated variants. As in scenario 1, all of the step-based methods had similar powers. In all three datasets, the power of the full model was higher than that of the step-based methods. For example, at 5% level of significance, the power for full model was 0.872 compared to power of 0.797 for the step-down-up method when the number of non-associated variants was 20 (see panel 1). In the first panel the proposed LDSEL method had higher power than all the step-based methods, VT, SKAT and also slightly better power than the full model. Once again, the step-based methods had lower power because of their inability to select variants in LD with their associated variants. The power for VT method was lower than the full model but higher than the step-based methods.

**Table 3:** Power comparison for different approaches at the 5% and 1% levels of significance.

Method	Power					
	20 NAV		70 NAV		170 NAV	
	5%	1%	5%	1%	5%	1%
Step-up	0.794	0.560	0.508	0.290	0.319	0.134
Full model	0.872	0.710	0.733	0.502	0.575	0.313
Step-down	0.797	0.566	0.511	0.292	0.322	0.135
Step-up-down	0.794	0.560	0.508	0.290	0.319	0.134
Step-down-up	0.797	0.566	0.511	0.292	0.322	0.135
LDSEL	0.882	0.715	0.794	0.590	0.629	0.361
SKAT	0.838	0.604	0.734	0.528	0.608	0.338
VT	0.824	0.596	0.635	0.373	0.504	0.226

The three panels correspond to simulation scenario 2 having 20, 70, and 170 non-associated variants (NAV) respectively along with three LD blocks of 10 variants, with 2 causal variants in each block.

In panel 2 and 3 of Table 3, we provide power comparisons when the number of non-associated variants (i.e. number of noisy variants) is 70 and 170, respectively. Once again, all the step-based methods had very similar power. In panel 2, the full model had a power of 0.733 which was higher than all the step-based methods. However, now the power of our proposed LDSEL method was higher than the full model, SKAT, and VT. Similar results were noted in panel 3.

Table 4 shows the power comparison results at the 5% and 1% levels of significance for the third scenario, in which the variants were simulated to be in linkage equilibrium with each other. The step-based methods had maximum powers of 0.469 and 0.237 at the 5% and 1% levels of significance, respectively. The full model had powers of 0.331 and 0.128 at the 5% and 1% levels of significance, respectively, which was considerably lower than that of the step-based methods. The VT method had significantly lower power than all the methods in this scenario. The LDSEL method had powers of 0.442 and 0.216 at 5% and 1% levels of significance, respectively, which was significantly higher than the full model but marginally lower than the step-based methods and SKAT.

**Table 4:** Power comparison for different approaches at the 5% and 1% levels of significance for simulation scenario 3, where all the variants were in linkage equilibrium.

Method	Power	
	5%	1%
Step-up	0.463	0.232
Full model	0.331	0.128
Step-down	0.469	0.237
Step-up-down	0.463	0.232
Step-down-up	0.469	0.237
LDSEL	0.442	0.216
SKAT	0.45	0.226
VT	0.172	0.074

## Discussion

In this paper, we proposed a novel methodology for detecting rare variants. Our motivation was to use the information about linkage disequilibrium between variants to select the best subset of rare variants to improve the power of association. The proposed subset selection algorithm can be incorporated seamlessly into existing methodologies that up-weigh the effect of rare alleles based on MAFs and also use binary weights, 1 or −1, depending upon the direction of the effect of the variant on the disease (i.e., deleterious or protective)[1]–[3], [6]–[8].

We simulated a range of scenarios for LD between the variants. We found that in most of the scenarios, our proposed LDSEL approach had powers higher than or comparable to those of the existing variable selection approaches. LDSEL is a flexible method and, depending on the structure of LD between the variants, it converts to a full model when all of the rare variants are in LD or to a step-based approach when all the variants are in linkage equilibrium. Applying LDSEL when the variants were in LD led to a gain in the power of association for the rare variants. The step-based subset selection methods had the highest power when the variants were not in LD.

The proposed method utilizes LD among variants to select the best subset of variants. There are several measures to assess linkage disequilibrium (e.g. 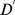, 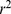). We used the 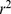 measure because it is commonly used in the context of genetic association studies [12]. It is possible that, for rare variants, the true LD may be over or under estimated using 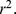 In our LDSEL approach, overestimation (underestimation) of LD would result in selecting more (less) rare variants in the model. In general the effect of error in estimation of LD on power depends on various factors, the true LD structure between the variants, the number of causal variants, and the minor allele frequencies of the variants.

The proposed LDSEL method utilized 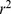 threshold of 0.1 to select the best subset. The threshold was selected based on assessing its impact on power in our simulation study. We performed power comparisons for different values of 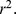 Specifically, we compared the results for three LD thresholds: 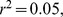 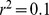 and 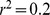. In general as the threshold decreases the proposed method is closer to the full model and as the threshold increases it is closer to the step-based methods. In the LDSEL method, we proposed a threshold of 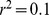 because it led to increased power in most of the scenarios considered in this manuscript.

As the number of non-associated variants being pooled increased, substantial power was gained by the LDSEL method compared with the full model or the step-based selection methods. We also simulated a range of odds ratios from 1.4 to 2 for the causal variants under scenario 2 with similar conclusions (data not shown). In general, no single method is uniformly powerful over all the scenarios and all the ranges of odds ratios. The computational burden for LDSEL is heavy, as is the case with the step-based selection methods and any permutation-based test. However, with increasing computation power, the time it takes to complete analyses using LDSEL is not prohibitive. Also, the computational burden can be alleviated by use of adaptive permutation methods. There is a need to leverage any possibility of gaining power for association, and this is even more important with rare variants, which are very hard to detect because of the limitations of sample size.

There is the possibility that some biologically important variants may be removed from methods that select rare variants because of the small sample size or because they are in linkage equilibrium. We recommend including all such important variants in our subset selection approach. Some of these rare variations may result in protein changes that negatively affect the protein function. Using the SIFT (Sorting Intolerant from Tolerant) program [13], one can predict whether a single-nucleotide polymorphism will affect protein function. There is a lot of potential for improving the methods of rare variant association analysis by using all the available data for these variants from pathways and from the variants that disrupt protein-coding functions.

In summary, our proposed method provides a flexible way to select a subset of variants for rare variant association analysis to improve the power of association, while using the information contained in the linkage disequilibrium between the variants. Last but not least, a better understanding of the linkage disequilibrium patterns in rare variants will help us develop novel and efficient ways to exploit the data.

## Supporting Information

Text S1 (DOCX)
