# Supplementary material for: A Linkage Disequilibrium–Based Approach to Selecting Disease-Associated Rare Variants
Source: PLoS One. 2013 Jul 11;8(7):e69226. doi: 10.1371/journal.pone.0069226 (PMC3708889; doi:10.1371/journal.pone.0069226)
Supplement: Text S1 — (DOCX) [file pone.0069226.s001.docx]

**Text S1.**

**Algorithms for step-based selection methods**

**For all the algorithms detailed below, assume the following parameters: K = Total number of variants pooled.**

**Algorithm for Step-up selection:**

1. **Select the best single variant model (i.e., best test statistic with a single variant in the model) out of the K models, i.e., for all** $j\in(1,2,\ldots,K)$ **find max(**$T^{j}$**). Let us denote this by** $T^{max}$**. Let** $x^{*}=x_{1}^{*}$ **denote the selected subset and** $x_{1}^{*}$ **denote the variant picked up in step 1.**
2. **Next, for all** $j\in\left(\left(1, 2, \ldots, K\right)-x^{*}\right)$ **find max(**$T^{j,x^{*}}$**). If max(**$T^{j,x_{ }^{*}}$**) >** $T^{max}$ **, then** $T^{max}$**= max(**$T^{j,x^{*}}$**) and** $x^{*}=(x_{1}^{*},x_{2}^{*})$ **.**
3. **Repeat step 2 until max(**$T^{j,x_{ }^{*}}$**) <** $T^{max}$**.**

**The final selected subset** $x^{*}$ **is the optimal subset using the step-up method.**

**Algorithm for Step-down selection:**

1. **Start with the full model in which all the variants are included**$x^{*}=(1,2,..,K)$**.**
2. **Select the best (K-1) variant model (i.e., best test statistic with K-1 variants in the model) out of the K models, i.e., for all** $j\in(1,2,\ldots,K)$ **find max(**$T^{x^{*}-j}$**). Let us denote this by** $T^{max}$**. Let** $x^{*}=(x^{*}-x_{1}^{*})$ **denote the selected subset and** $x_{1}^{*}$ **denote the variant dropped in step 1.**
3. **Next, for all** $j\in(x^{*})$**, find max(**$T^{x^{*}-j}$**). If max(**$T^{x_{ }^{*}-j}$**) >** $T^{max}$ **, then** $T^{max}$**= max(**$T^{x^{*}-j}$**) and** $x^{*} =(x^{*}-x_{2}^{*}).$
4. **Repeat step 3 until max(**$T^{x^{*}-j}$**) <** $T^{max}$**.**

**The final selected subset** $x^{*}$ **is the optimal subset using the step-down method.**

**Algorithm for Step-up-down selection:**

1. **Start with the null model with no variants included**$x^{*}=()$**.**
2. **Perform step-up selection for 1 step.**
3. **Perform step-down selection for 1 step.**
4. **Repeat step 2 and step 3 alternately until max(**$T^{x^{*},j}$**) <** $T^{max}$**.**

**The final selected subset** $x^{*}$ **is the optimal subset using the step-up-down method.**

**Algorithm for Step-down-up selection:**

1. **Start with the full model with all the variants included**$x^{*}=(1,2,\ldots,K)$**.**
2. **Perform step-down selection for 1 step.**
3. **Perform step-up selection for 1 step.**
4. **Repeat step 2 and step 3 alternately until max(**$T^{x^{*},j}$**) <** $T^{max}$**.**

**The final selected subset** $x^{*}$ **is the optimal subset using the step-down-up method.**
